# Novel diagnostic and prognostic biomarkers of colorectal cancer: Capable to overcome the heterogeneity-specific barrier and valid for global applications

**DOI:** 10.1371/journal.pone.0256020

**Published:** 2021-09-02

**Authors:** Yasir Hameed, Muhammad Usman, Shufang Liang, Samina Ejaz

**Affiliations:** 1 Department of Biotechnology, Institute of Biochemistry, Biotechnology and Bioinformatics, The Islamia University of Bahawalpur, Bahawalpur, Pakistan; 2 State Key Laboratory of Biotherapy and Cancer Center, West China Hospital, Sichuan University and Collaborative Innovation Center for Biotherapy, Chengdu, 610041, P.R. China; 3 Department of Biochemistry, Institute of Biochemistry, Biotechnology and Bioinformatics, The Islamia University of Bahawalpur, Bahawalpur, Pakistan; University of Oklahoma Health Sciences Center, UNITED STATES

## Abstract

**Introduction:**

The heterogeneity-specific nature of the available colorectal cancer (CRC) biomarkers is significantly contributing to the cancer-associated high mortality rate worldwide. Hence, this study was initiated to investigate a system of novel CRC biomarkers that could commonly be employed to the CRC patients and helpful to overcome the heterogenetic-specific barrier.

**Methods:**

Initially, CRC-related hub genes were extracted through PubMed based literature mining. A protein-protein interaction (PPI) network of the extracted hub genes was constructed and analyzed to identify few more closely CRC-related hub genes (real hub genes). Later, a comprehensive bioinformatics approach was applied to uncover the diagnostic and prognostic role of the identified real hub genes in CRC patients of various clinicopathological features.

**Results:**

Out of 210 collected hub genes, in total 6 genes (CXCL12, CXCL8, AGT, GNB1, GNG4, and CXCL1) were identified as the real hub genes. We further revealed that all the six real hub genes were significantly dysregulated in colon adenocarcinoma (COAD) patients of various clinicopathological features including different races, cancer stages, genders, age groups, and body weights. Additionally, the dysregulation of real hub genes has shown different abnormal correlations with many other parameters including promoter methylation, overall survival (OS), genetic alterations and copy number variations (CNVs), and CD8+T immune cells level. Finally, we identified a potential miRNA and various chemotherapeutic drugs via miRNA, and real hub genes drug interaction network that could be used in the treatment of CRC by regulating the expression of real hub genes.

**Conclusion:**

In conclusion, we have identified six real hub genes as potential biomarkers of CRC patients that could help to overcome the heterogenetic-specific barrier across different clinicopathological features.

## 1. Introduction

Colorectal cancer (CRC) is the most common cancer and is one of the leading causes of cancer-related deaths worldwide [1]. Genetic mutations and altered expression levels of various tumor suppressor genes including Adenomatous Polyposis Coli (APC), Tumor Protein 53 (TP53) and BReast CAncer gene (BRCA1) have long been considered as an important cause of CRC, which affect the development, progression, and metastasis of CRC through a variety of regulatory pathways [2].

Advances in biomarkers screening technologies have greatly helped to discover the reliable novel diagnostic and prognostic biomarkers for the early detection and treatments of CRC. However, due to the heterogeneity-specific nature of the available diagnostic and prognostic biomarkers the recurrence and metastasis of CRC in patients of different races, different cancer stages, genders, age groups, and body weight are still, not fully addressed and remain the major challenge to clinical treatment [3,4].

In the recent times, microarray technology has become very popular among the scientist as it enables them to screen thousands of differentially expressed messenger RNAs (mRNAs), microRNAs (miRNAs), and long noncoding RNAs (lncRNAs) together [5–7], which play an important role in the development and progression of a disease. Besides, this technology has also helped to perform the in-depth analyses of the key genes to explore potential molecular targets and diagnostic biomarkers [8].

The Gene Expression Omnibus database (GEO, available at: http://www.ncbi.nlm.nih.gov/geo/) is an open-access gene expression database, which is created, and maintained by the National Center for Biotechnology Information (NCBI) [9,10]. This database is used to store and freely distribute the microarrays, next generation sequencing, and various other forms of high-throughput functional genomic datasets to the researchers worldwide [10].

GEO database is the most attractive platform for the researchers to re-evaluate and re-analyze the microarray datasets through different bioinformatics approaches for the identification of disease-specific novel potential biomarkers. Many researchers have previously utilized the CRC microarray expression datasets to identify the potential biomarkers as hub genes. However, by considering the fact that, biomarkers are highly race, cancer stage, genders, age, and body weight-specific biomolecules and knowing that CRC microarray expression datasets, available in the GEO database, contain information extracted from patients of different races, different cancer stages, genders, age groups, and body weights. Moreover, on daily basis new datasets are added to the GEO database and each dataset suggests different CRC related hub genes. Hence, it has become clinically impossible to globally employ the hub genes which have been reported by individual studies through extensive analysis of specific GEO’s-CRC related microarray expression datasets [11] as potential diagnostic and prognostic biomarkers to the CRC patients of all the races, cancer stages, genders, age groups, and body weights. Therefore, in this study, we planned to re-analyze the already reported CRC-related hub genes through a multi-layered bioinformatics based strategy to detect few more closely CRC-linked hub genes (real hub genes) that could commonly be used as potential diagnostic and prognostic biomarkers for CRC patients of different clinicopathological features.

For this purpose, the already reported hub genes will be extracted from those published studies that utilized the CRC-related GEO expression datasets. Then, all these hub genes will be added to a single pool to establish a consolidated set of most significantly dysregulated hub genes exhibiting the high degree of centrality among the analyzed genes. Followed by this step, the pool of hub genes will be subjected to pathway enrichment analysis, PPI network construction and identification of the most centralized genes (real hub genes) and their underlying pathways [12]. Next, the differential expression and validation analysis of the identified real hub genes in normal and CRC patients of different clinicopathological features will be carried out via multiple authentic platforms such as GEPIA database [13], GENT2 database, UALCAN database [14] and cBioPortal database [15] using numerous TCGA colon adenocarcinoma (COAD) datasets consisting of a large cohort of normal individuals and COAD patients. Following this, we will investigate the correlation of the real hub genes expressions with their promoter methylation level, genetic alterations, copy number variations (CNVs), overall survival (OS) and CD8+ T immune cells levels in CRC patients relative to normal controls. Additionally, in an attempt to understand the regulatory mechanisms, miRNAs interaction patterns will be studied to explore the role of miRNAs, if any, as mediators of the real hub genes’ expression behavior. Similarly the effect of various chemotherapeutic drugs on expression profile of the identified real hub genes will be deduced through hub genes-drug interaction network analysis. The information obtained could help to regulate the real hub genes expression during the treatment of CRC.

Taken together, this detailed mega-scale study based upon information retrieved from the analysis of large number of datasets and reported hub genes is therefore expected to find some common CRC biomarkers which can be exploited for the diagnostic and prognostic purposes of worldwide CRC patients of different clinicopathological features and thus helpful to overcome the heterogenetic-specific barrier. The information retrieved can further help in predicting the treatment outcomes in CRC patients.

## 2. Material and methods

### 2.1 Literature search and hub genes extraction

A PubMed based search was performed to search all the studies which analyzed the CRC expression microarray datasets available on GEO (available at: https://www.ncbi.nlm.nih.gov/geo/) database and identified the hub genes. For this purpose, two keywords “Hub genes AND colon cancer” and “Hub genes AND colorectal cancer” were searched on PubMed separately with the “Original article” filter. In total 108 studies appeared which were further explored to filter out the studies having desired information. In total, 21 studies were selected which were found to collectively analyze more than 30 CRC expression based microarray datasets retrieved from the GEO database and identify numerous hub genes. We extracted all the hub genes from these studies and assembled them in the form of a single pool.

### 2.2 Pathway enrichment analysis

The Database for Annotation, Visualization and Integrated Discovery (DAVID, available at: https://david.ncifcrf.gov) integrates biological data as well as analytical tools to systematically and comprehensively annotate the biological functions [16]. We used the DAVID database for pathway enrichment analysis of the pooled hub genes. A p-value < 0.05 indicated the statistically significant differences.

### 2.3 Protein–protein interaction (PPI) network construction and mining the real hub genes

The protein–protein interaction (PPI) analysis is important to interpret the molecular mechanisms of the key pathways in carcinogenesis. In the present study, we utilized the Search Tool for the Retrieval of Interacting Genes (STRING) database (available at: https://string-db.org/) [17] to construct the PPI network of all the pooled hub genes. The six real hub genes present in the PPI network were then identified through Cytohubba application of the Cytoscape tool (version:3.7.1) [18], which can explore important nodes and fragile motifs in a network by several topological algorithms including degree-edge percolated component and degree of centrality.

### 2.4 GEPIA dataset analysis

GEPIA (available at: http://gepia.cancer-pku.cn/) is an online platform of retrieved data from the UCSC Xena database (available at: https://xena.ucsc.edu/), which in-houses the expression data of 9736 tumor samples and 8587 normal samples [13]. In this study, the transcriptional expression levels of the real hub genes were analyzed in COAD patients relative to control. For this purpose the Colon adenocarcinoma (COAD) dataset was utilized which includes 275 tumor and 349 normal samples. A t-test is used for the statistics purpose in GEPIA. The expression level of real hub genes in GEPIA was normalized as transcript per million (TPM) reads, and a p-value < 0.05 was considered to be statistically significant. We also utilized this database for the correlational analysis between real hub genes expression and OS duration of the COAD patients.

### 2.5 GENT2 dataset analysis

Gene Expression database of Normal and Tumor tissues 2 (GENT2, available at: http://gent2.appex.kr) is an online platform that provides a user-friendly overview of the gene expression patterns across different normal and tumor tissues compiled from publically available GEO datasets. GENT2 contains the expression data of more than 68,000 samples and has several useful functions. For example, GENT2 provides gene expression analysis option across 72 different cancerous tissues. GENT2 also provides an option to study the differential expression and its prognostic significance based on tumor subtypes. Additionally, GENT2 provides a meta-analysis of survival information to provide users more reliable prognostic value of a gene of interest. A t-test is used for the statistics purpose in GENT2. In the present study, this platform was used for further validation of the GEPIA based results of real hub genes expression patterns examined in COAD patients relative to controls [19]. The expression level of real hub genes in GENT2 was normalized as transcript per million (TPM) reads, and a p-value of < 0.05 was considered to be statistically significant.

### 2.6 UALCAN dataset analysis

The UALCAN (available at; http://ualcan.path.uab.edu/) is an online publicly available web-portal that offers in-depth analysis of data from TCGA. In the present study, this database was used for the genes promoters’ methylation analysis and validation of variations detected in the real hub genes’ mRNA and protein expression profiles of COAD patients of different clinicopathological features relative to normal controls. In UALCAN t-test was used for the statistics purpose. The mRNAs’ expression levels of real hub genes were normalized as transcript per million (TPM) reads. While corresponding proteins expression levels were normalized as z-value, the promoters’ methylation levels were normalized as beta (β) value, and a p-value of < 0.05 value was considered to be statistically significant.

### 2.7 cBioPortal analyses

An open-source tool, cBioPortal (Available at: http://www.cbioportal.org) developed by the Computation Biology Center located at Sloan Kettering, was utilized to summarize all the possible transcriptional changes, mutual expression tendencies, and overall survival through Kaplan-Meier analysis, by presenting the results as OncoPrint. In this study, the cBioPortal database was used to analyze genetic variations such as (amplifications, deep deletions, and mutations) in the real hub genes in COAD patients.

### 2.8 Real hub genes and infiltrating level of CD8+ T cells in COAD patients

TIMER (available at: https://cistrome.shinyapps.io/timer/) is a web resource for systematical evaluations of the clinical impact of different immune cells in diverse cancer types [20]. In the present study, this database was used to find the Spearman correlation between the levels of real hub genes’ expression and CD8+ T immune cells. In TIMER a t-test was used for the statistics purpose and a p-value of < 0.05 was considered to be statistically significant.

### 2.9 The miRNA–real hub gene interaction network analysis

The miRNAs, targeting the real hub genes, were predicted through miRNA target prediction databases “Regulatory Network Repository of Transcription Factor and microRNA Mediated Gene Regulations (RegNetwork) database” (available at: http://regnetworkweb.org/). The RegNetwork database contains information of experimentally validated regulatory elements of gene expression including transcription factors (TFs) and miRNAs. This platform provides a user-friendly interface for the submission of query of interest and allows the finding of combinatorial and synergic regulatory relationships among TFs, miRNAs and genes [21]. A co-expression network based on the correlation analysis of real hub genes and miRNAs associated with the cancer was then developed by Cytoscape software. In the network, interaction between the miRNAs and real hub gene was represented by an arrow. The numbers of arrows in the networks indicated the contribution of one miRNA in the expression regulation of the surrounding genes.

### 2.10 Real hub gene-drug interaction network analysis

The Comparative Toxicogenomics Database (CTD, available at: http://ctdbase.org/) has been employed to obtain the information of chemotherapeutic drugs that could reduce or enhance the mRNAs or proteins expression levels of the genes of interest [22]. Briefly, all the real hub genes were searched in the CTD database, and hub gene-drug interaction networks were visualized using Cytoscape software.

## 3. Results

### 3.1 Literature search and hub genes extraction

In total 21 studies were selected, some of them have identified the hub genes in individual microarray dataset of CRC [23–25] while others have used the combination of multiple microarray datasets of CRC [26–28]. We extracted all hub genes reported in literature and pooled them after normalizing the duplicated genes, hence, a pool of 210 hub genes from 31 microarray datasets, containing 3128 CRC and 877 normal samples, were selected for further analysis (Table 1). Raw data without normalization can be seen in S1 Table (Supporting Information).

**Table 1:** Details of CRC microarray based expression datasets and the identified hub genes.

Dataset	No. samples C/N	Source of origin	Extracted hub genes	References
GSE17538 GSE29623 GSE81558 GSE21510 GSE44076 GSE24514 GSE32323 GSE110225 GSE20916 GSE73360 GSE44861 GSE23878 GSE41258 GSE110224 GSE35279 GSE87211 GSE117606 GSE28000 GSE21815 GSE75970 GSE39582 GSE9348 GSE22598 GSE113513 GSE74602 GSE101502 GSE2509 GSE7621 GSE14333 GSE4183 GSE4107	244/0 130/0 42/9 148/0 98/148 37/12 28/16 30/30 108/37 62/30 111/111 35/24 328/62 17/17 74/5 230/133 102/102 86/26 123/9 8/0 585/0 70/12 18/18 14/14 30/30 3/3 6/0 16/9 290/0 45/8 10/12	USA USA Spain Japan Spain Finland Japan Greece Poland Italy USA Saudi Arabia Israel Greece Japan USA Belgium USA Japan China France Singapore Japan China Singapore China Italy USA Australia Hungary Singapore	HCLS1, EVI2B, CD48, DSN1, ALDH1A1, TUBAL3, RRM2, SHMT2, PGM1, CCT6A, IDH3A, HSPA2, UGP2, RUVBL1, CDK1, CCNB1, MAD2L1, AHCY, HSPH1, BUB1B, DARS, KIF18A, NUF2, CENPF, ERCC6L, SPC25, CXCL1, CXCL12, SST, NPY, PPY, LPAR1, CCL19, GNG4, CXCL5, CXCL2, CXCL3, NPY1R, CCL28, TIMP1, SPP1, MMP3, GCG, KIT, BMP2, COL1A1, CXCL8, CXCL11, NMU, PPBP, COL3A1, TGFR1, CD44, MMP1. SPARC, MYC, COL1A2, CA2, SERPINB5, CCND1, PTGS2, MET, GDF15, ECT2, CKAP2, CSE1L, ABCE1, RFC3, TPx2, SLC4A4, SLC26A3, CLCA4, SLC25A2, CEACAM7, PDGFRB, FZD2, PRKCB, FPR2, KIF2C, KIF20A, DLGAP5, NCAPG, UBE2C, EXO1, CDC45, CXCL10, DTL, PF4, CEP55, P2RY14, ADNP, CDK4, CEBPB, CENPA, CENPH, CENPN, RFC2, SSTR1, SSTR2, HCAR3, APLN, CXCR2, SAA1, PMCH, GAL, CXCR1, CCL23, CXCL6, HTR1D, GALR1, CNR1, AGT, FPR1, PTGDR2, CCR8, INSL5, F2RL2, GCC, GRP, OXTR, GPR4, NPSR1, UTS2B, PROK2, AGTR1, EDN3, CHRM1, DK1, CCNA2, PLK1, KIF11, MELK, NUSAP1, MCM4, RFC4, PTTG1, CHEK1, CENPE, ITGA2, BGN, SULF1, FA, THBS2, CTHRC1, COL5A2, AQP8, CLCA4, GUCA2B, MS4A12, GUCA2A, ABCG2, CLDN8, ZG16, PKIB, CA4, BEST4, CA1, MT1M, CD177, HSD17B2, ADH1C, CLCA1, FOXQ, KRT23, LY6G6D, MMP7, CDH3, CST1, CRNDE, DPEP1, EPHX4, CLDN1, CEL, CLDN2, SLC35D3, COL11A1, SLCO1B3, CKMT2, NUDT21, GNB1, CLINT1,EGFR, HRas, Akt1, CDKN1a, PCNA, NAT1, NAT2, PLAGL2, POFUT1,TOP2A, ACLY, VEDFA, GMPS, ENO1, ACTA2, AURKA, CDC42, TEX11, QKI, CAV, FN1, ARHGEF6, JUP, WNT2, WNT5A, WNT11, PYY, CCL20	[11,23–25,27–43]
**Total =** 31	**Total =** 3128/877		**Total =** 210	

**C** = Cancerous, **N** = Normal, USA = United States of America.

### 3.2 KEGG pathway analysis of the pooled hub genes

**Fig 1:**
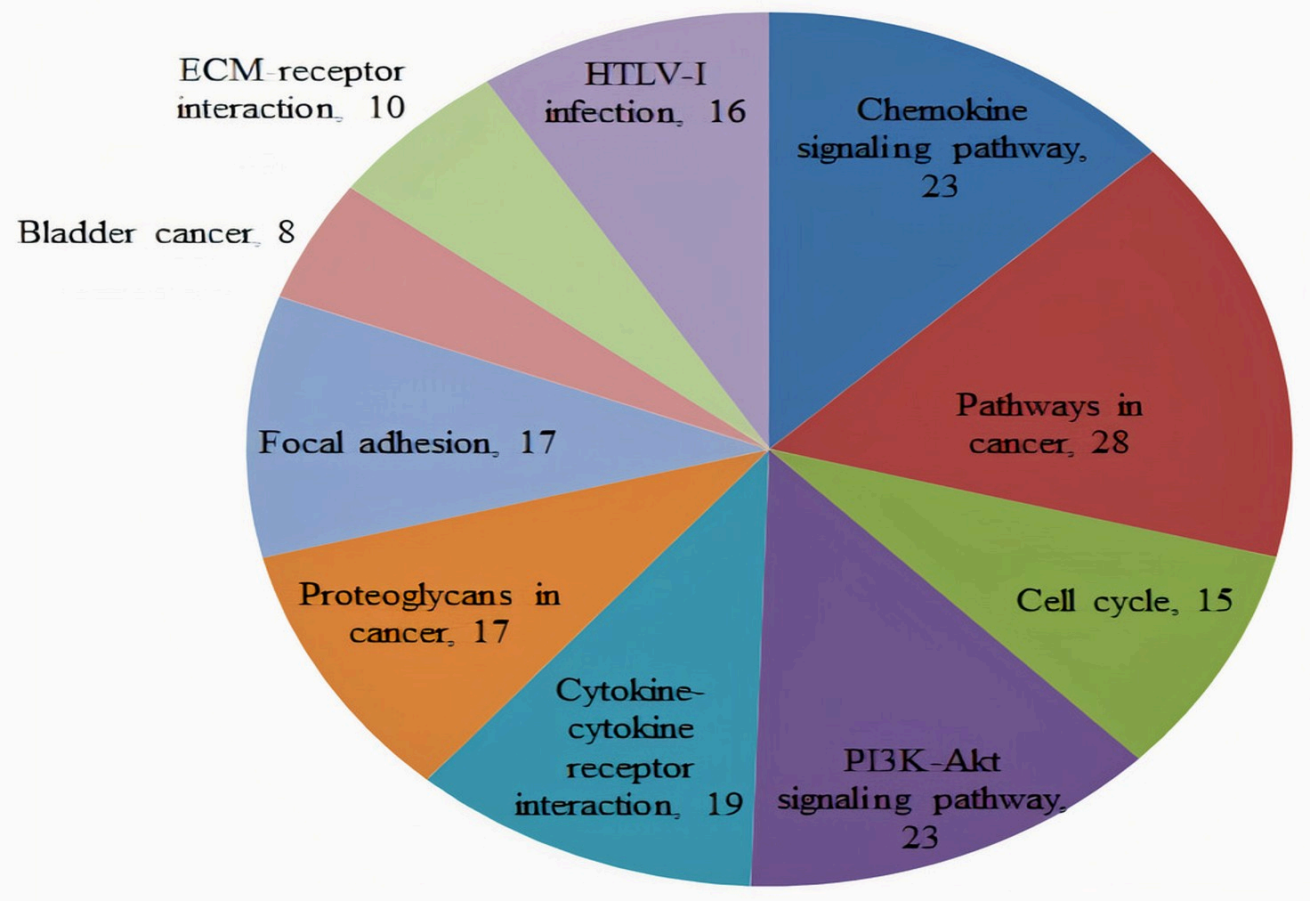
A heatmap of the KEGG pathways to portray role of all the CRC related hub genes (n = 210) identified during present study.

KEGG pathway enrichment analysis suggested that all the 210 pooled hub genes were significantly enriched in pathways including ‘Chemokine signaling pathway’, ‘Pathways in cancer’, ‘Cell cycle’, ‘PI3K-Akt signaling pathway’, and ‘Cytokine-cytokine receptor interaction pathway’. The Top 10 KEGG terms related to the hub genes are enlisted in Fig 1 and Table 2).

**Table 2:** KEGG pathway analysis details of the 210 pooled hub genes, extracted from the various GEO microarray CRC expression datasets.

Pathway ID	Pathway Name	Gene count	p-value	Gene name
04062	Chemokine signaling pathway	23	<0.05	CXCL1, HRAS, CXCL5, CXCL3, CXCL2, CXCR1, CXCL8, CCL19, CXCR2, PF4, CXCL6, CXCL11, CCL28, CXCL12, CXCL10, AKT1, CDC42, CCR8, CCL23, PPBP, CCL20, GNB1, GNG4
05200	Pathways in cancer	28	<0.05	WNT5A, HRAS, PTGS2, CXCL8, LPAR1, KIT, CXCL12, MMP1, WNT2, AKT1, AGTR1, CDC42, GNG4, MYC, FN1, EGFR, BMP2, MET, ITGA2, FZD2, CDK4, PRKCB, JUP, CDKN1A, CCND1, GNB1, PDGFRB, WNT11
04110	Cell cycle	15	<0.05	CDK1, CHEK1, PTTG1, CDK4, MCM4, CCNB1, CDC45, CDKN1A, CCND1, MAD2L1, PLK1, PCNA, BUB1B, MYC, CCNA2
04151	PI3K-Akt signaling pathway	23	<0.05	EGFR, HRAS, COL3A1, MET, ITGA2, LPAR1, KIT, CDK4, COL5A2, AKT1, CCND1, CDKN1A, GNB1, CHRM1, COL1A2, PDGFRB, COL1A1, GNG4, THBS2, MYC, COL11A1, SPP1, FN1
04060	Cytokine-cytokine receptor interaction	19	<0.05	CXCL1, BMP2, CXCL5, CXCL3, CXCL2, CXCR1, CXCL8, CCL19, CXCR2, PF4, CXCL6, CXCL11, CCL28, CXCL12, CXCL10, CCR8, CCL23, PPBP, CCL20
05205	Proteoglycans in cancer	17	<0.05	EGFR, WNT5A, HRAS, HCLS1, MET, ITGA2, FZD2, PRKCB, AKT1, WNT2, CDC42, CDKN1A, CCND1, CD44, WNT11, MYC, FN1
04510	Focal adhesion	17	<0.05	EGFR, HRAS, MET, COL3A1, ITGA2, COL5A2, PRKCB, AKT1, CDC42, CCND1, COL1A2, PDGFRB, COL1A1, THBS2, COL11A1, SPP1, FN1
05219	Bladder cancer	8	<0.05	EGFR, CDKN1A, HRAS, CCND1, CXCL8, CDK4, MYC, MMP1
04512	ECM-receptor interaction	10	<0.05	CD44, COL3A1, COL1A2, ITGA2, COL1A1, COL11A1, THBS2, COL5A2, SPP1, FN1
05166	HTLV-I infection	16	<0.05	WNT5A, HRAS, CHEK1, PTTG1, FZD2, CDK4, WNT2, AKT1, CDKN1A, CCND1, MAD2L1, PCNA, PDGFRB, BUB1B, WNT11, and MYC

### 3.3 PPI network construction, real hub genes selection and their pathway enrichment

**Fig 2:**
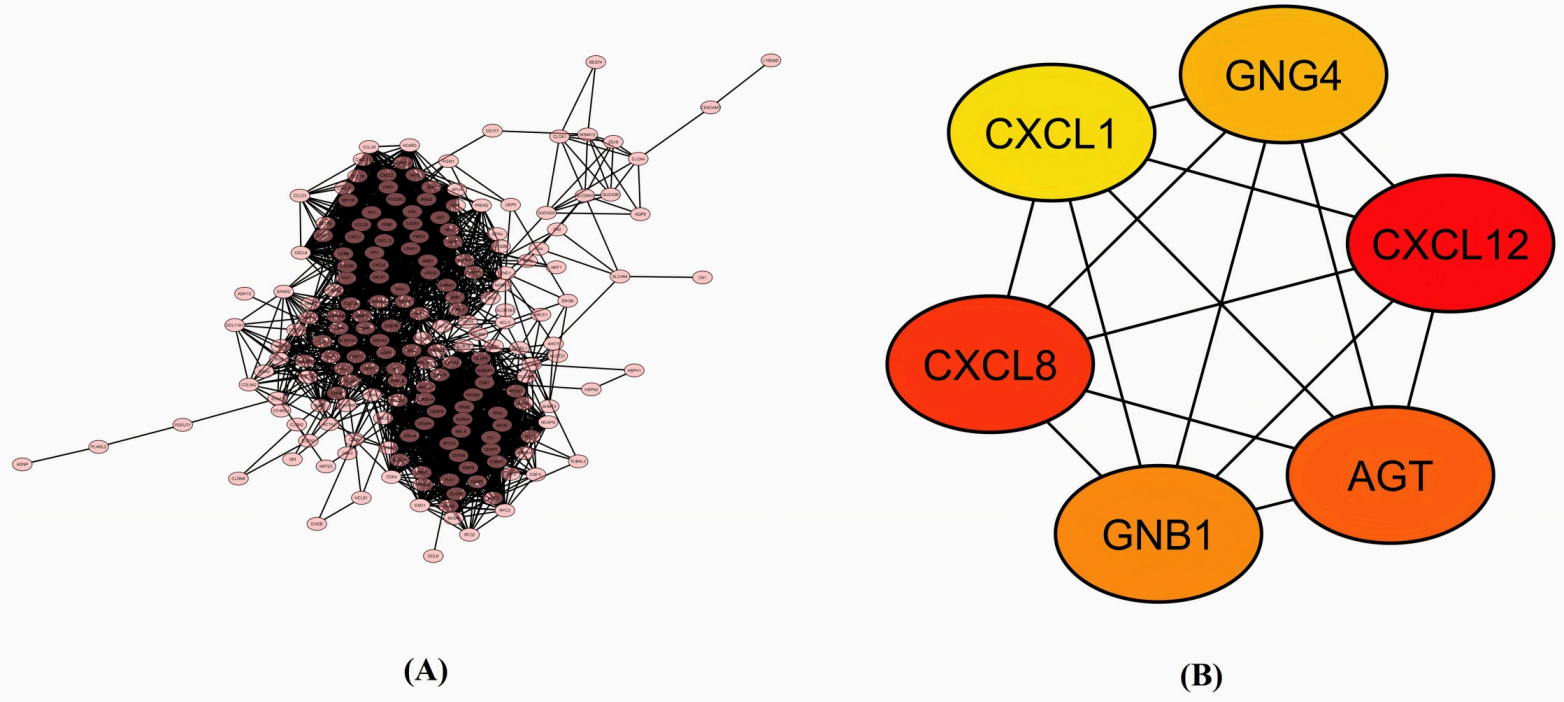
**(A) A** PPI network of all the 210 extracted hub genes. **(B)** A network of six real hub genes identified on the basis of degree of centrality.

A PPI network of all the 210 pooled hub genes was constructed through an online available STRING database containing 204 nodes and 2561 edges. A Cytohubba analysis was carried out through Cytoscape software to identify more closely CRC relevant few genes (real hub genes). Based on the degree of centrality, the group of the identified real six hub genes included CXCL12, CXCL8, AGT, GNB1, GNG4, and CXCL1 (Fig 2 and Table 3).

**Table 3:** List of the real hub genes identified from the PPI network of the extracted 210 CRC related hub genes.

Sr. No	Name of the gene	Degree of centrality	No. Nodes	Closeness of centrality
1	CXCL12	68	68	0.50
2	CXCL8	66	66	0.53
3	AGT	61	61	0.47
4	GNB1	60	60	0.49
5	GNG4	59	59	0.47
6	CXCL1	56	56	0.47

**Degree of centrality =** It is the number of links incident upon a node.

**Closeness of centrality =** It is a measure of the average shortest distance from one node to other node.

Furthermore, the pathway enrichment analysis of the identified real hub, using David tool, has shown the significant (p<0.05) involvement of five genes in “Chemokine signaling pathway”, “Pathways in cancer”, and “Cytokine-cytokine receptor interaction” pathways (Table 4).

**Table 4:** Details of the KEGG pathway analysis of the identified real hub genes.

Pathway ID	Pathway Name	Gene count	p-value	Gene name
04062	Chemokine signaling pathway	05	<0.05	CXCL1, GNB1, CXCL8, GNG4, CXCL12
05200	Pathways in cancer	04	<0.05	GNB1, CXCL8, GNG4, CXCL12
04060	Cytokine-cytokine receptor interaction	03	<0.05	CXCL1, CXCL8, CXCL12

### 3.4 Bioinformatics based expression analysis of the identified real hub genes in normal individuals and colon adenocarcinoma (COAD) patients

In order to analyze and validate the differential mRNA expression of the identified real hub genes in normal and COAD patients of different clinicopathological features (different race, cancer stages, genders, age groups, and body weights), a detailed bioinformatics analysis was carried out. For the said purpose we considered three different online available platforms including, GEPIA; it retrieved mRNA data from UCSC Xena server which contained 275 COAD samples paired with 349 normal samples and used to analyze the mRNA expression of the real hub genes in the present study, GENT2; it retrieved mRNA data from GEO database which contained 477 COAD samples paired with 91 normal samples and utilized to validate the real hub genes expression in the present study, and finally the UALCAN; it retrieved mRNA data from TCGA database which contained 286 COAD samples paired with 41 normal samples and used to validate the real hub genes expression at protein level (in overall COAD cases relative to controls) and at mRNA level (in COAD patients of different clinopathological features relative to controls).

**Fig 3:**
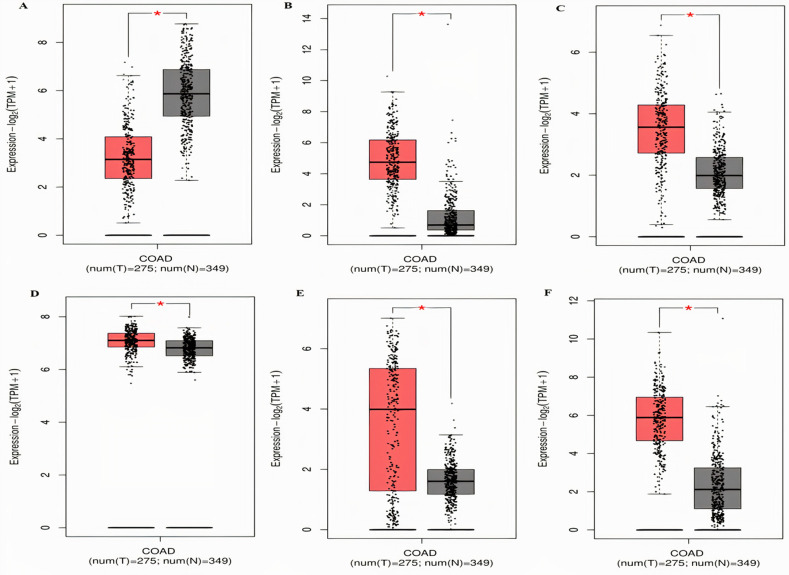
Box plots showing the relative mRNA expression levels of real hub genes in normal and COAD patients (Information retrieved via GEPIA database). The box plots shows the relative mRNA expression of: **(A)** CXCL12, **(B)** CXCL8, **(C)** AGT, **(D)** GNB1, **(E)** GNG4, and **(F)** CXCL1; in COAD patients and normal samples. A p-value of <0.05 was considered significant.

**Fig 4:**
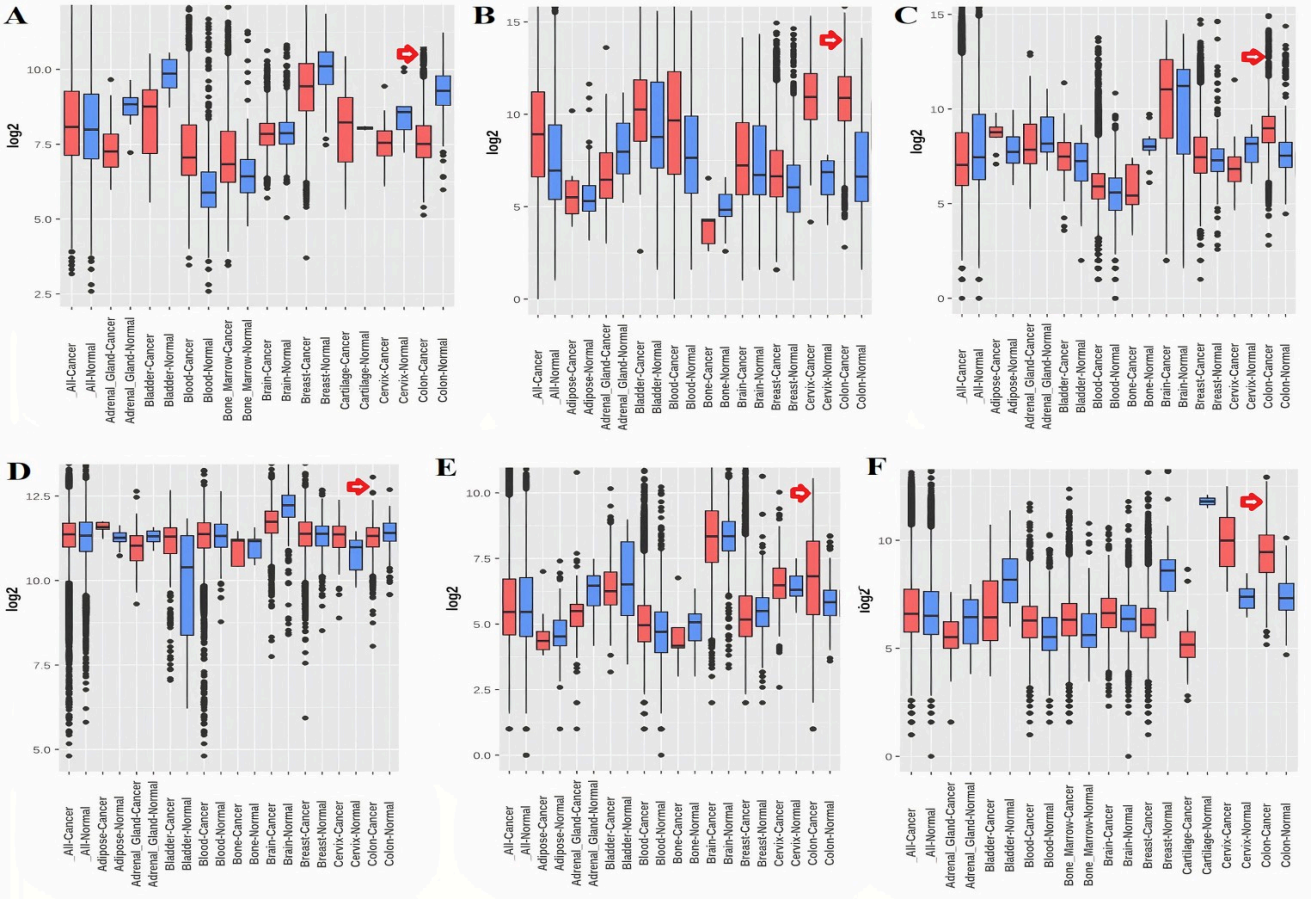
Box plots showing the relative expression levels of real hub genes in normal and COAD patients (Information retrieved from GENT2 database). The box plots shows the relative mRNA expression of: **(A)** CXCL12, **(B)** CXCL8, **(C)** AGT, **(D)** GNB1, **(E)** GNG4, and **(F)** CXCL1; in COAD patients and normal samples. A p-value of <0.05 was considered significant.

**Fig 5:**
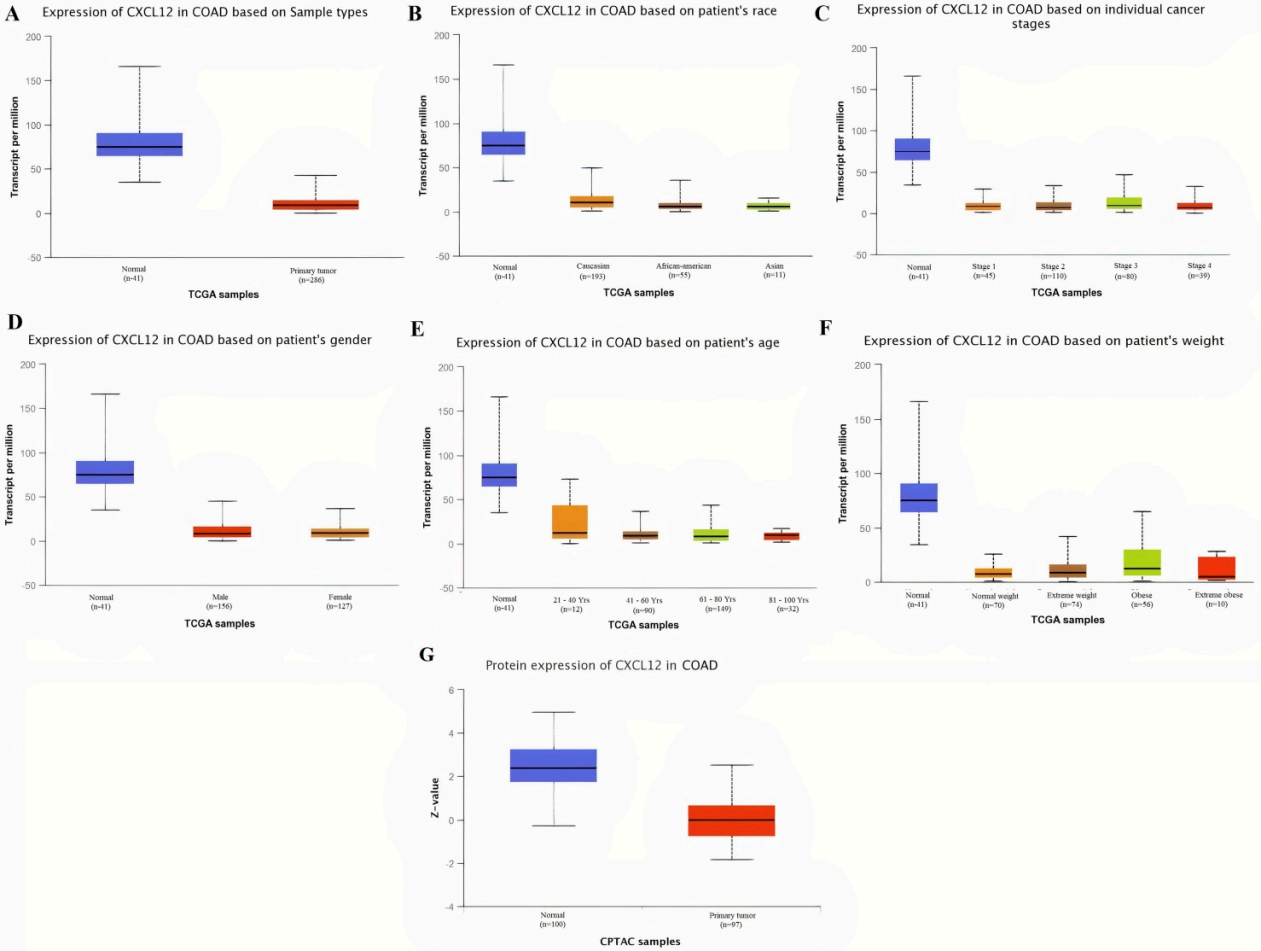
Box plots showing the relative expression levels of CXCL12 in normal and COAD samples of different clinicopathological features via UALCAN database. Relative mRNA expression of CXCL12: **(A)** in normal individuals and COAD patients; **(B)** in normal individuals and COAD patients of different races; **(C)** in normal individuals and COAD patients of different cancer stages; **(D)** in normal individuals and COAD patients of different genders; **(E)** in normal individuals and COAD patients of different age groups; and, **(F)** in normal individuals and COAD patients of different body weights; **(G)** Relative protein expression of CXCL12 in normal individuals and COAD patients. A p-value of <0.05 was considered significant. Normal weight = BMI greater than or equal to 18.5 and BMI less than 25, Extereme weight = BMI greater than or equal to 25 and BMI less than 30, Obese = BMI greater than or equal to 30 and BMI less than 40, and Extreme Obese = BMI greater than 40.

**Fig 6:**
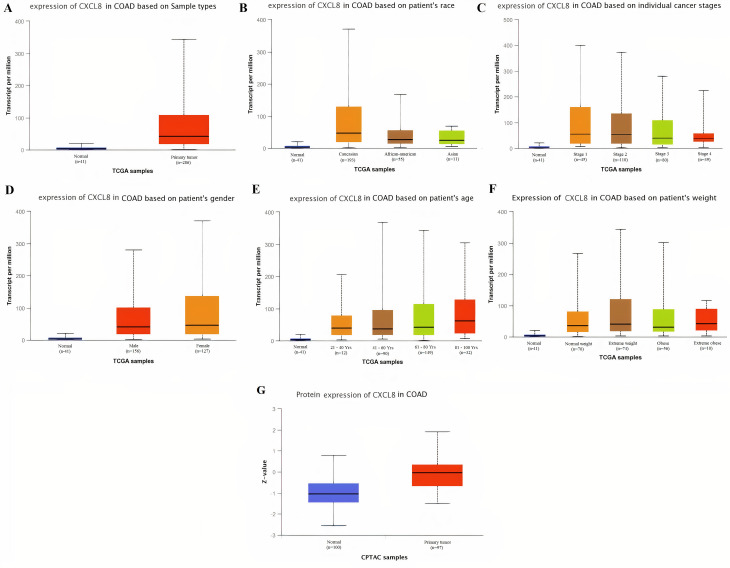
Box plots showing the relative expression levels of CXCL8 in normal and COAD samples of different clinicopathological features via UALCAN database. Relative mRNA expression of CXCL8: **(A)** in normal individuals and COAD patients; **(B)** in normal individuals and COAD patients of different races; **(C)** in normal individuals and COAD patients of different cancer stages; **(D)** in normal individuals and COAD patients of different genders; **(E)** in normal individuals and COAD patients of different age groups; and, **(F)** in normal individuals and COAD patients of different body weights; **(G)** Relative protein expression of CXCL8 in normal individuals and COAD patients. A p-value of <0.05 was considered significant. Normal weight = BMI greater than or equal to 18.5 and BMI less than 25, Extereme weight = BMI greater than or equal to 25 and BMI less than 30, Obese = BMI greater than or equal to 30 and BMI less than 40, and Extreme Obese = BMI greater than 40.

**Fig 7:**
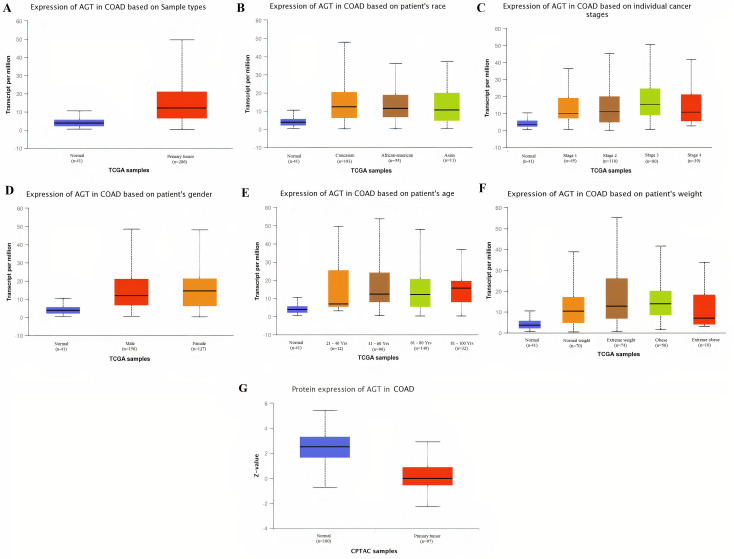
Box plots showing the relative expression levels of AGT in normal and COAD samples of different clinicopathological features via UALCAN database. Relative mRNA expression of AGT: **(A)** in normal individuals and COAD patients; **(B)** in normal individuals and COAD patients of different races; **(C)** in normal individuals and COAD patients of different cancer stages; **(D)** in normal individuals and COAD patients of different genders; **(E)** in normal individuals and COAD patients of different age groups; and, **(F)** in normal individuals and COAD patients of different body weights; **(G)** Relative protein expression of AGT in normal individuals and COAD patients. A p-value of <0.05 was considered significant. Normal weight = BMI greater than or equal to 18.5 and BMI less than 25, Extereme weight = BMI greater than or equal to 25 and BMI less than 30, Obese = BMI greater than or equal to 30 and BMI less than 40, and Extreme Obese = BMI greater than 40.

**Fig 8:**
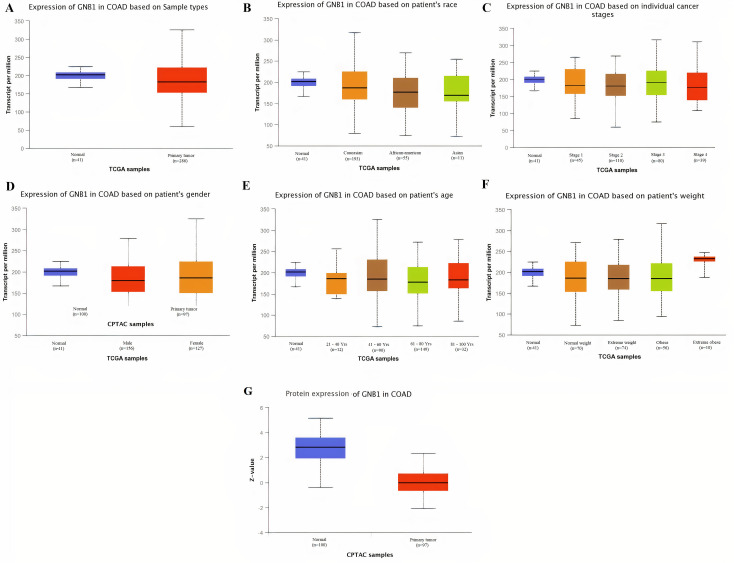
Box plots showing the relative expression levels of GNB1 in normal and COAD samples of different clinicopathological features via UALCAN database. Relative mRNA expression of GNB1: **(A)** in normal individuals and COAD patients; **(B)** in normal individuals and COAD patients of different races; **(C)** in normal individuals and COAD patients of different cancer stages; **(D)** in normal individuals and COAD patients of different genders; **(E)** in normal individuals and COAD patients of different age groups; and, **(F)** in normal individuals and COAD patients of different body weights; **(G)** Relative protein expression of GNB1 in normal individuals and COAD patients. A p-value of <0.05 was considered significant. Normal weight = BMI greater than or equal to 18.5 and BMI less than 25, Extereme weight = BMI greater than or equal to 25 and BMI less than 30, Obese = BMI greater than or equal to 30 and BMI less than 40, and Extreme Obese = BMI greater than 40.

**Fig 9:**
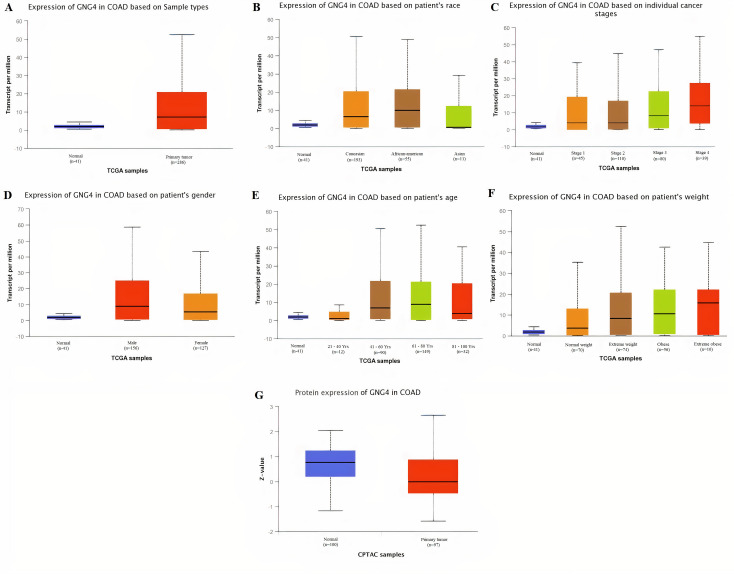
Box plots showing the relative expression levels of GNG4 in normal and COAD samples of different clinicopathological features via UALCAN database. Relative mRNA expression of GNG4: **(A)** in normal individuals and COAD patients; **(B)** in normal individuals and COAD patients of different races; **(C)** in normal individuals and COAD patients of different cancer stages; **(D)** in normal individuals and COAD patients of different genders; **(E)** in normal individuals and COAD patients of different age groups; and, **(F)** in normal individuals and COAD patients of different body weights; **(G)** Relative protein expression of GNG4 in normal individuals and COAD patients. A p-value of <0.05 was considered significant. Normal weight = BMI greater than or equal to 18.5 and BMI less than 25, Extereme weight = BMI greater than or equal to 25 and BMI less than 30, Obese = BMI greater than or equal to 30 and BMI less than 40, and Extreme Obese = BMI greater than 40.

**Fig 10:**
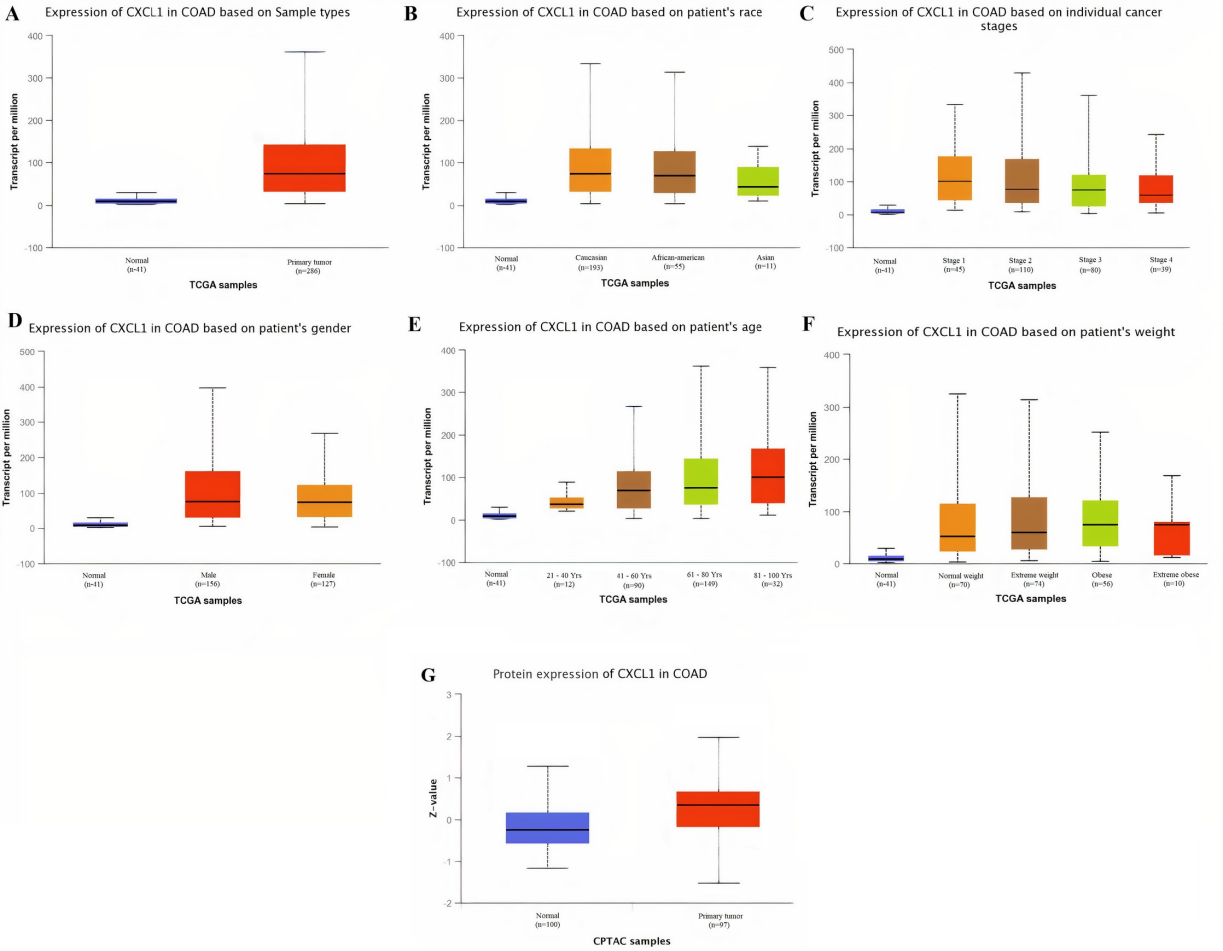
Box plots showing the relative expression levels of CXCL1 in normal and COAD samples of different clinicopathological features via UALCAN database. Relative mRNA expression of CXCL1: **(A)** in normal individuals and COAD patients; **(B)** in normal individuals and COAD patients of different races; **(C)** in normal individuals and COAD patients of different cancer stages; **(D)** in normal individuals and COAD patients of different genders; **(E)** in normal individuals and COAD patients of different age groups; and, **(F)** in normal individuals and COAD patients of different body weights; **(G)** Relative protein expression of CXCL1 in normal individuals and COAD patients. A p-value of <0.05 was considered significant. Normal weight = BMI greater than or equal to 18.5 and BMI less than 25, Extereme weight = BMI greater than or equal to 25 and BMI less than 30, Obese = BMI greater than or equal to 30 and BMI less than 40, and Extreme Obese = BMI greater than 40.

Taken together the results of these three databases, we observed and validated the significant (p<0.05) down-regulation of CXCL12 while significant (p<0.05) overexpression of CXCL12, CXCL8, AGT, GNB1, GNG4, and CXCL1 real hub genes at mRNA level in COAD patients of different clinicopathological features (patients race, cancer stages, genders, age groups, and body weight) relative to the normal controls. Moreover, at protein level, UALCAN based results confirmed the significant (p<0.05) overexpression of CXCL8, GNG4, and CXCL1 while the insignificant (p>0.05) down-regulation of CXCL12, AGT, and GNB1 (Figs 3–10). This scenario of inverse correlation between mRNA and protein expression levels of AGT, and GNB1 indicates the abnormal posttranscriptional regulation which probably decreases the half-life of these proteins and results in their down-regulation.

### 3.5 Promoters methylation levels of the real hub genes in normal individuals and COAD patients

**Fig 11:**
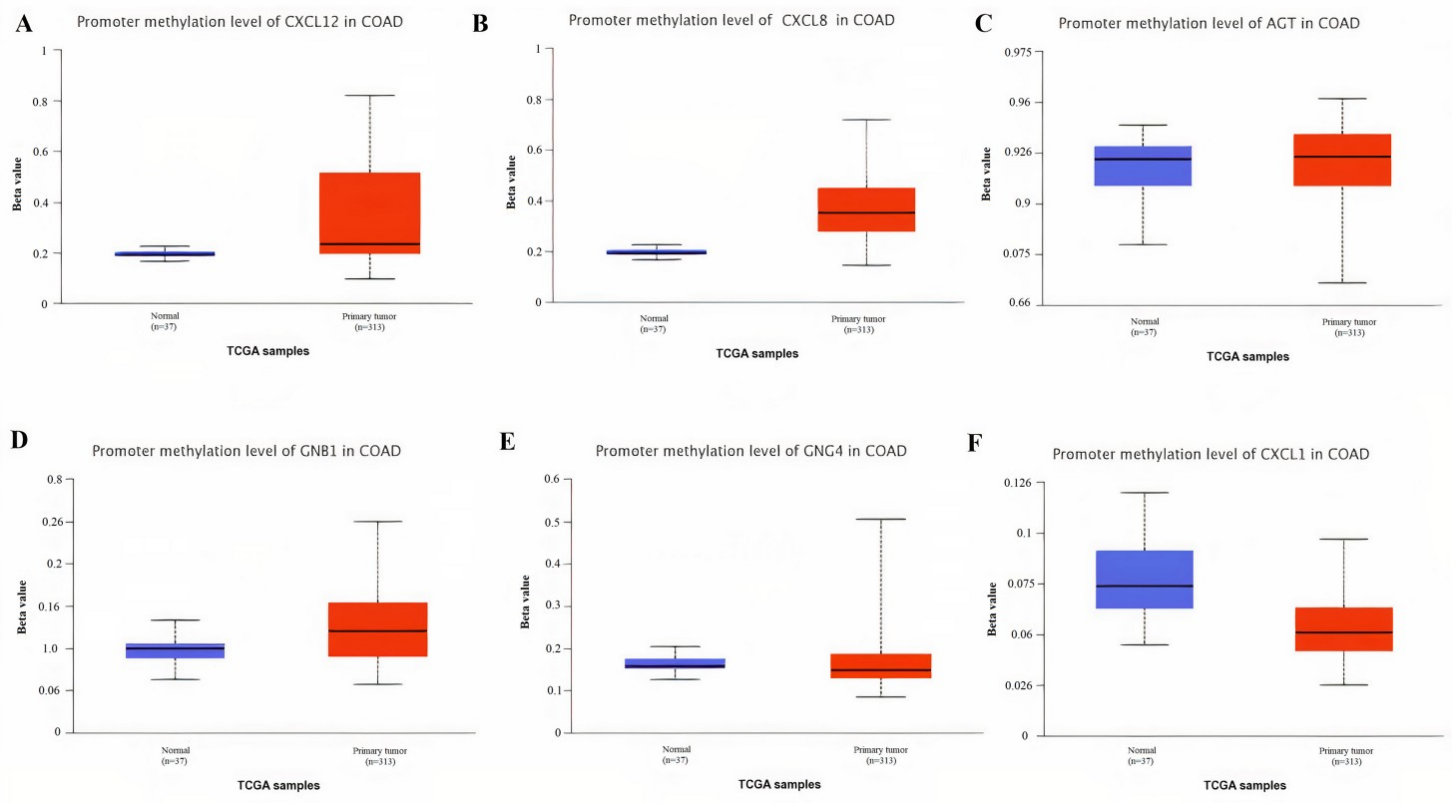
Box plot showing real hub genes promoter methylation levels in normal individuals and COAD patients via UALCAN database. The box plot shows the promoter methylation levels of: (**A**) CXCL12 **(B)** CXCL8 **(C)** AGT **(D)** GNB1 **(E)** GNG4 **(F)** CXCL1 in normal individuals and COAD patients. A p-value of <0.05 was considered significant.

The variations in the degree of promoter’s methylation have regulatory impact on the expression behavior of the onco and proto-onco genes and thus have been closely linked with cancer development [44]. To document the variations, if any, examined in COAD patients as compared to the normal controls we studied the promoters’ methylation levels of the real hub genes via UALCAN platform containing information of 13 normal and 313 COAD samples. A diverse pattern of real hub genes promoters’ methylation levels were observed in COAD patients relative to normal controls. Approximately 30%, 20%, 60%, 57%, 56%, and 55% COAD patients have had the similar promoter methylation levels of CXCL12, CXCL8, AGT, GNB1, GNG4, and CXCL1, respectively, as examined in case of the normal controls. Moreover, 15%, 10%, 20%, 15%, 24%, and 45% COAD patients were found positive for the hypomethylation of CXCL12, CXCL8, AGT, GNB1, GNG4, and CXCL1 promoters, respectively, relative to normal controls. While the remaining 55%, 70%, 20%, 38%, 20%, and 0% COAD patients were found positive for the hypermethylation of CXCL12, CXCL8, AGT, GNB1, GNG4, and CXCL1 promoters, respectively, relative to normal controls. Ultimately, the overall comparison between COAD and normal groups by applying the t-test revealed that CXCL12, CXCL8, AGT, GNB1, and GNG4) were significantly (p<0.05) hypermethylated while CXCL1 was significantly (p<0.05) hypomethylated in COAD samples relative to normal controls (Fig 11). Collectively, these results suggested that promoter methylation level positively correlates with the mRNA expression level of CXCL8, AGT, GNB1, and GNG4 while negatively correlates with the mRNA expression level of CXCL1 and CXCL12 in COAD samples.

### 3.6 Amplification, deletion, mutation, and fusion of real hub genes in COAD

**Fig 12:**
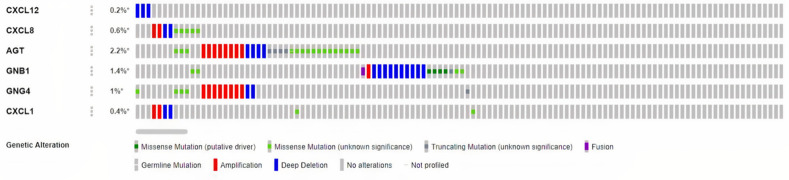
Frequency of the genetic alterations and CNVs of the real hub genes in COAD patients.

Genetic alteration and CNVs are the common sources of gene expression dysregulation [45]. In this study, information related to the real hub genes-related genetic alterations and CNVs information were obtained from three different TCGA COAD datasets (TCGA firehose legacy, TCGA nature 2012 and TCGA PanCancer Atlas containing data of 619, 266 and 87 COAD samples, respectively), via cBioPortal platform. Results of the analysis revealed the varying degrees of genetic alterations and CNVs among all the real hub genes in 1482 analyzed COAD samples. Out of which AGT displayed the highest incidence rate of genetic alterations and CNVs (2.2%, 32/1482) followed by the GNB1 which showed the incidence rate of 1.4% (21/1482). Following GNB1, the other real hub genes including GNG4, CXCL8, CXCL1, and CXCL12 showed the 1% (15/1484), 0.6% (15/1482), 0.4% (6/1482) and, and 0.2% (3/1482) incidence rate of genetic alterations and CNVs in COAD samples (Fig 12). In CXCL8 and AGT, missesnse mutations accounted for most of the changes, while in CXC12 and GNB1, deep deletions were identified as the most frequent changes. Moreover, in case of GNG4 and CXCL1 deep amplifications were highlighted as the most common changes in the queued samples.

### 3.7 Prognostic values of the real hub genes in the COAD patients

**Fig 13:**
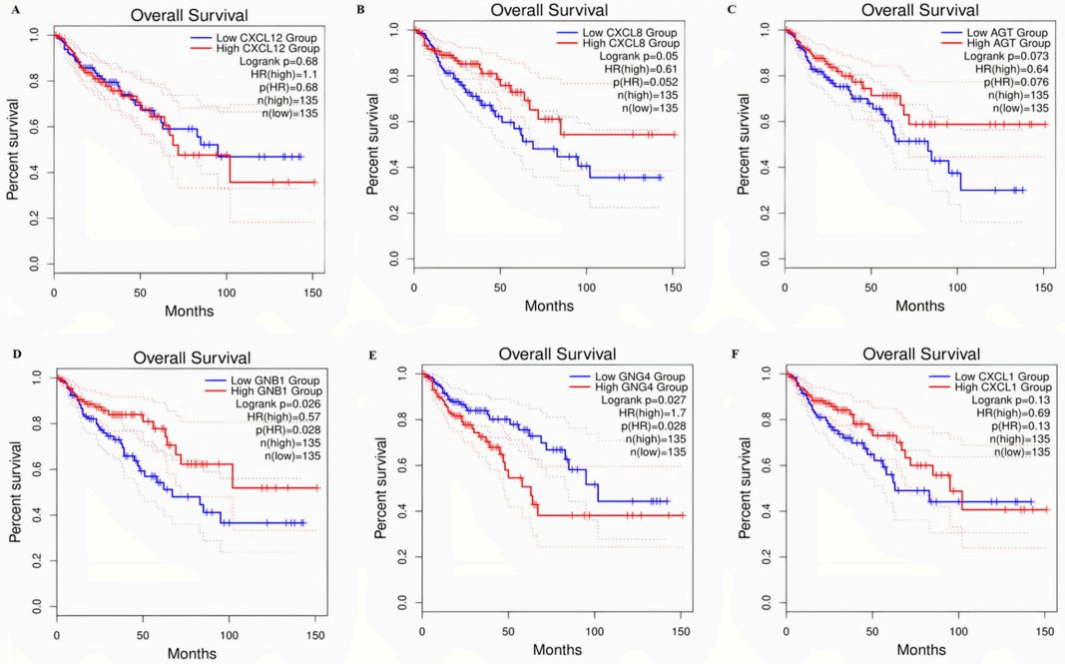
The COAD related prognostic information of the real hub genes obtained via GEPIA database. The calculated prognostic value of: **(A)** CXCL12, **(B)** CXCL8, **(C)** AGT, **(D)** GNB1, **(E)** GNG4, **(F)** CXCL1 gene. Blue color indicates this low expression while red color indicates the high expression of a gene.

Correlation analysis among the mRNA’s expression levels of the real hub genes and the overall survival (OS) of the COAD patients was performed via GEPIA database and using data of 275 normal and 349 COAD samples. Results revealed that the higher mRNAs’ expression levels of GNG4 [HR: 1.7, p>0.05] are significantly correlated with the reduced OS duration of the COAD patients, therefore, suggested as good prognostic biomarker for predicting the OS duration of the COAD patients. Results also revealed that down-regulation of CXCL12 [HR: 1.1, p>0.05] and up-regulations of CXCL8 [HR: 0.61, p>0.05], AGT [HR: 0.64, p>0.05], GNB1 [HR: 0.57, p<0.05], and, CXCL1 [HR: 0.69, p<0.05] are the bad prognostic biomarkers for predicting the OS duration of the COAD patients as they do not correlated with the reduced OS duration (Fig 13).

### 3.8 Real hub genes and infiltrating levels of CD8+ T cells in COAD patients

**Fig 14:**
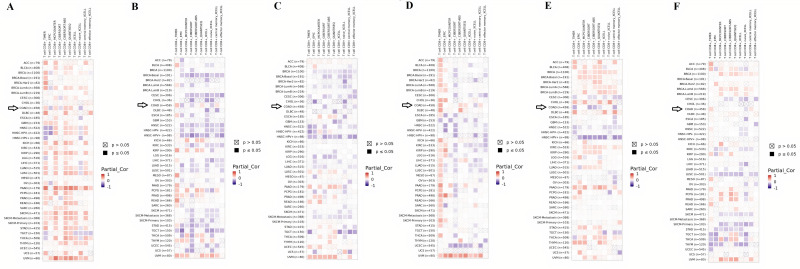
TIMER based Spearman correlational analysis between the expression of real hub genes and CD8+ T immune cell infiltration in COAD. TIMER based Spearman correlational analysis between: **(A)** CXCL12 expression and CD8+ T immune cell infiltration; **(B)** CXCL8 expression and CD8+ T immune cells’ infiltration; **(C)** AGT expression and CD8+ T immune cells’ infiltration; **(D)** GNB1 expression and CD8+ T immune cells’ infiltration; **(E)** GNG4 expression and CD8+ T immune cells’ infiltration; **(F)** CXCL1 expression and CD8+ T immune cells’ infiltration. Red color box represents the positive correlation; blue color represents the negative correlation while white color represents no correlation. A p-value of <0.05 was considered as significant.

The functions of, and interactions between, the innate and adaptive immune systems are vital for the anticancer immunity. Cytotoxic T cells expressing cell-surface CD8 are the most powerful effectors in the anticancer immune response and form the backbone of current successful cancer immunotherapies [46]. In the current study, the Spearman correlation between the expression of real hub genes and CD8+ T cell infiltration was calculated using TIMER database. Results revealed a significant (p>0.05) positive correlation between the mRNA expression of the CXCL12, GNB1 and CXCL1 and CD8+ T immune cells’ infiltration while a significant (p>0.05) negative correlation between the mRNA expression of CXCL8, AGT and GNG4 and CD8+ T immune cells’ infiltration (Fig 14).

### 3.9 Investigation of miRNAs-real hub genes interaction network

**Fig 15:**
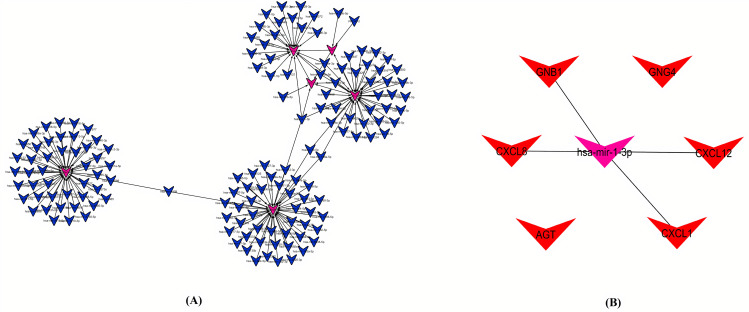
The miRNA–real hub gene interaction network. The pink circular node represents the miRNA. The blue v shape node represents the hub gene while pink and red v shape node represents the mir-1-3p/CXCL8 or CXCL12, CXCL1, and GNB1 axis. The arrow shape represents the interaction between the miRNAs and real hub genes.

In this study, the RegNetwork database was used for predicting the miRNAs targeting real hub genes to investigate the regulatory relationships between real hub genes and miRNAs. The co-expression network was developed by Cytoscape software and was based on the correlation analysis between the hub genes and miRNAs (Fig 15). The numbers of miRNAs and mRNAs in the network were 200 and 6, respectively. In the miRNA-real hub gene network, the numbers of arrows indicates the contribution of each miRNA in the expression regulation of the surrounding real hub genes. The miRNA-real hub gene interaction analysis findings revealed that mir-1-3p targets the most (four) of the genes including CXCL8, CXCL12, CXCL1, and GNB1. The miR-1-3p has been widely proven as a tumor suppressor [47]. In CRC, contradicting reports were found reporting expression variations in miR-1-3p. Mainly, miR-1-3p was found down-regulated in different studies investigating its association with CRC [47–49]. However, one exceptional study has also reported its up-regulation [50]. In view of our findings, we speculate that four real hub genes (CXCL8, CXCL12, CXCL1, and GNB1) function as an oncogenes and down-regulation of mir-1-3p may lead to the up-regulation of these genes as a different axis (mir-1-3p/CXCL8 or CXCL12, CXCL1, and GNB1) in the pathogenesis of CRC. Therefore, it will be worth to therapeutically target the mir-1-3p/CXCL8, or CXCL12, CXCL1, and GNB1 axis in the treatment of CRC (Fig 15).

### 3.10 Real hub genes-drug interaction network analysis

**Fig 16:**
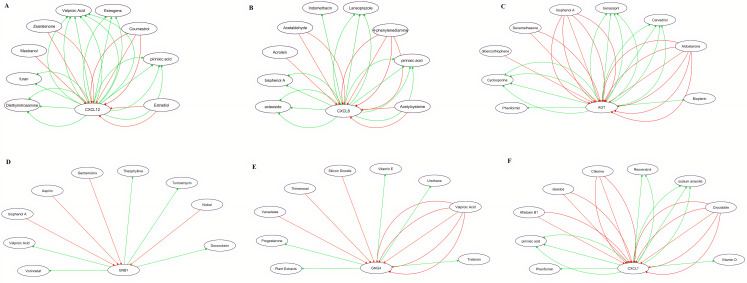
Real hub gene-drug interaction network of the identified real hub genes. Panels A–F indicates the available chemotherapeutic drugs in CTD that decrease or increase the expression levels of the real hub genes. **(A)** Real hub gene-drug network of CXCL12; **(B)** Real hub gene-drug network of CXCL8; **(C)** Real hub gene-drug network of AGT; **(D)** Real hub gene-drug network of GNB1; **(E)** Real hub gene-drug network of GNG4; **(F)** Real hub gene-drug network of CXCL1. Red arrows indicate the chemotherapeutic drugs that could increase the expression levels of the real hub genes, while green arrows indicate the chemotherapeutic drugs that could decrease the expression levels of the real hub genes. The numbers of arrows between chemotherapeutic drugs and real hub genes in the network represent the supported numbers of previous studies reported in literature.

In order to explore the relationship between real hub genes and available cancer therapeutic drugs, a gene-drug interaction network was developed using CTD database and analyzed via Cytoscape. In view of the gene-drug interaction network analysis results, it was observed that mRNA expression of the identified real hub genes including CXCL12, CXCL8, AGT, GNB1, GNG4 and CXCL1 could potentially be influenced by a variety of drugs. For example, valporic acid and furan could reduce the expression levels of CXCL12 while estradiol and coumestrol could elevate CXCL8 expression levels (Fig 16).

## 4. Discussion

CRC is the most common type of gastrointestinal tumor and one of the leading causes of cancer-related deaths worldwide [26]. Although recent advances in CRC screening and treatment approaches have proven helpful in the management of disease, its worldwide prevalence is on a rise due to the heterogeneity-specific nature of the available biomarkers used for its detection and monitoring the prognosis [3,4]. It is, therefore, crucial to uncover the molecular mechanisms underlying CRC initiation, development, and progression for the identification of some novel diagnostic and prognostic biomarkers that could be used for detection and monitoring the prognosis and treatment of CRC over the heterogeneity-specific barrier.

In the present study, we conducted a PubMed based search to identify all the studies which utilized GEO-based CRC microarray expression datasets to explore the hub genes. In total 21 studies were identified which collectivity utilized more than 30 CRC microarray expression datasets from the GEO. We extracted all the identified hub genes from each of the studies and pooled them to make a set of 210 hub genes representing all the CRC microarray expression datasets utilized by these studies. Then, we performed the pathway enrichment and PPI network construction and analysis of the extracted hub gens to identify the few more closely CRC-related genes (real hub genes) and their underlying pathways. We further performed the expression analysis and validation of the real hub genes, and correlation analysis of their expressions with their promoter methylation and OS survival duration of the COAD patients through a comprehensive multi-layered bioinformatics approach. We also aimed to identify the genetic alterations and to perform the miRNA-real hub genes interaction network analysis and real hub genes-drug interaction network analysis of the identified six real hub genes.

KEGG pathway enrichment analysis revealed that all the extracted 210 hub genes were significantly enriched in various pathways including ‘Chemokine signaling pathway’, ‘Pathways in cancer’, ‘Cell cycle’, ‘PI3K-Akt signaling pathway’, and Cytokine-cytokine receptor interaction pathway (Fig 1 and Table 2). Furthermore, PPI network of the extracted 210 hub genes illustrated the overview of their functional connections, of which top 6 real hub genes were selected as CXCL12, CXCL8, AGT, GNB1, GNG4 and CXCL1.

KEGG pathway analysis of the 6 real hub genes demonstrated that these genes were significantly (p<0.05) enriched in pathways including “Chemokine signaling pathway”, “Pathways in cancer”, and “Cytokine-cytokine receptor interaction”. In a broader sense, all these pathways basically represent the “Cytokine-cytokine receptor interaction” pathway because cytokine is a general term used for all signaling molecules while chemokine are the specific cytokines that function by attracting cells to sites of infection/inflammation and this pathway also belongs to the category of the “Pathways in cancer”.

Inflammation is an essential component of the tumor microenvironment and one of the hallmarks of cancer [51]. Chemokine’s, are a family of small, secreted, and structurally related cytokines with a crucial role in inflammation and immunity [52]. They are also key mediators of cancer-related inflammation being present at the tumor site for pre-existing chronic inflammatory conditions but also being the target of oncogenic pathways [53]. Initially, chemokine’s were identified playing a prominent role in determining the composition of tumor stroma, where they were found to directly affect the cancer cell proliferation and metastasis [54,55].

The identified real hub gene CXCL12 (stromal cell-derived factor 1) is an extracellular chemokine, which binds to CXCR4, a G-protein coupled receptor (GPCR) and have been well-recognized as a factor involved in the cancer metastasis [56]. The CXCL12 is normally secreted by Kuppfers and endothelial cells in the liver, which is the most common site for CRC metastases [57]. Previously, various studies have suggested that overexpression of CXCR4 is correlated with the poor survival and liver metastasis in CRC [58,59]. In addition, the elevated expression level of CXCR4 has also been observed in hypoxia due to the activity of hypoxia-inducible factor 1-α [60]. CXCR7 is another receptor that interacts with CXCL12 in CRC cells. Earlier, *Wang et al*. [61] assessed the expression of CXCL12, CXCR4, and CXCR7 in CRC and found that the expression of both CXCL12 and CXCR7 were significantly up-regulated in CRC samples as compared to controls [62]. Contrary to this, however, in the present study, we demonstrated the unusual significant (p<0.05) down-regulation of CXCL12 at mRNA levels in COAD patients of different clinicopathological features including different races, cancer stages, genders, age groups, and body weights as compared to the normal controls. Our results also showed the down-regulation of CXCL12 at translational level in COAD patients as compared to controls. We also investigated the correlation of CXCL12 down-regulation with its promoter methylation status and genetic alterations. In view of the results, it was noticed that significant (p<0.05) hyper methylation is the possible cause of CXCL12’s down-regulation in COAD rather than the genetic alterations which were observed in a least count (0.2%) of the analyzed samples. Taken together, our data suggested CXCL12’s down-regulation as a novel potential diagnostic biomarker in CRC patients of different races, cancer stages, genders, age groups, and body weights.

The real hub gene CXCL8, also known as neutrophil-activating factor (NAF), and interleukin 8 (IL-8), was the first chemokine identified as a leukocyte chemo-attractant [63]. CXCL8 controls the leukocyte trafficking during homeostasis and inflammation by interacting with (GPCR) receptor CXCR1 [64]. Many previous studies [65–67] have already demonstrated its up-regulation in CRC patients relative to controls, however, none of these studies generalized CXCL8 in CRC patients of different clinicopathological features (different races, cancer stages, genders, age groups, and body weights). But in our study, we observed and generalized the significant higher mRNA expression of CXCL8 in COAD patients of different clinicopathological features including different races, cancer stages, genders, age groups, and body weights as compared to the normal controls. Our results also showed CXCL8’s up-regulation at translation level in COAD patients with respect to controls. We further revealed that CXCL8 was significantly hyper methylated in COAD patients group relative to controls and also genetically altered in a least proportion (0.6%) of the COAD samples. Regarding unexpected hyper methylation, our findings contradict the commonly accepted association of hyper methylation and down regulated gene expression; therefore, we recommend further in-depth research to explore the clear role of promoter methylation level in the expression regulation of CXCL8. Collectively, our data suggested CXCL8 up-regulation as a novel potential diagnostic biomarker in CRC patients of different races, cancer stages, genders, age groups, and body weights.

The real hub gene ATG (Angiotensinogen) is an essential component of the renin-angiotensin system (RAS) which is a potent regulator of blood pressure. ATG is a precursor of Angiotensin-II (A-II) and mainly produced in hepatocytes [68]. So far, a single study has been found in published scientific literature reporting overexpression of AGT in CRC [69], however, this study lacks the information regarding clinicopathological features-specific expression variations in AGT. In the current study, we observed the significant (p<0.05) higher expression of ATG at mRNA levels in COAD patients of different clinicopathological features including different races, cancer stages, genders, age groups, and body weights as compared to the normal controls. In contrast to this, the results of present study also reported the lower expression of AGT at translation level in COAD patients relative to controls. This inverse correlation between AGT mRNA and translation levels indicates the defect in post-translation modifications which might reduce the half-life of AGT protein and results in its reduction. Our results further reported that AGT was significantly hyper methylated in COAD patients’ group than the controls and also genetically altered in a small proportion (2.2%) of the COAD samples. Regarding a positive correlation between AGT mRNA expression and hyper methylation, we recommend further in-depth research to explore the clear role of promoter methylation levels in the expression regulation of AGT. Overall, the results of our study suggested ATG’s up-regulation as a novel potential diagnostic biomarker in CRC patients of different races, cancer stages, genders, age groups, and body weights.

The real hub gene GNB1 encodes for Gβ_1_ which is a beta (β) subunit of the guanine nucleotide–binding protein that forms heterotrimeric complexes with G protein subunits α and γ. The Gβ subunit is joined to Gγ subunit to form a Gβγ complex which activates the RAS pathway, a signaling pathway responsible to maintain cell proliferation, cell adhesion, and cell migration [70]. Best to our knowledge, no study has yet reported GNB1 expression variation in CRC. However, one study has demonstrated its down-regulation in Clear-cell renal cell carcinoma (ccRCC) patients [71]. In the present study, we observed the significant (p<0.05) up-regulation of GNB1 mRNA in COAD patients of different clinicopathological features including different races, cancer stages, genders, age groups, and body weights as compared to the normal controls. Our results also showed the down-regulation of GNB1 at translation level in COAD patients relative to controls. This inverse correlation between GNB1 mRNA and translation levels indicates the abnormalities in post-translation modification mechanisms which might reduce the half-life of GNB1 protein and results in its reduction. We further revealed that GNB1 was significantly hyper methylated in group of COAD patients relative to control group and also genetically altered in a least proportion (1.4%) of the COAD samples. Regarding unexpected hyper methylation, our findings challenges the classical concept of methylation, therefore, further work is required to be done to get a more detailed view of correlation between expression and methylation of GNB1 in COAD. Taken together, our results suggested that GNB1 up-regulation may be considered as a novel potential diagnostic biomarker in CRC patients of different races, cancer stages, genders, age groups, and body weights.

The real hub gene GNG4 encodes for the γ subunit of the G protein trimmer which potentially functions as a positive regulator of the RAS pathway that is responsible to maintain cell proliferation, cell adhesion, and cell migration [72]. Best to our knowledge, nothing has been reported in references regarding its expression variations in CRC, however, one earlier study [73] has evaluated its down-regulation in glioblastoma (GBM). Present study demonstrated that GNG4 was significantly (p<0.05) overexpressed at mRNA level in CRC patients of different races, cancer stages, genders, age groups, and body weights. Results of this study also demonstrated the up-regulation of GNG4 at translation level in COAD patients relative to controls. We further reported that GNG4 was significantly hyper methylated in COAD patients group than the controls and also genetically altered in a small proportion (1%) of the COAD samples. This scenario of GNG4 overexpression and hyper methylation challenges the classical view where hyper methylation is always related with the down-regulation. In total, the results of our study suggested GNG4’s up-regulation as a novel potential diagnostic biomarker in CRC patients of different races, cancer stages, genders, age groups, and body weights.

**Fig 17:**
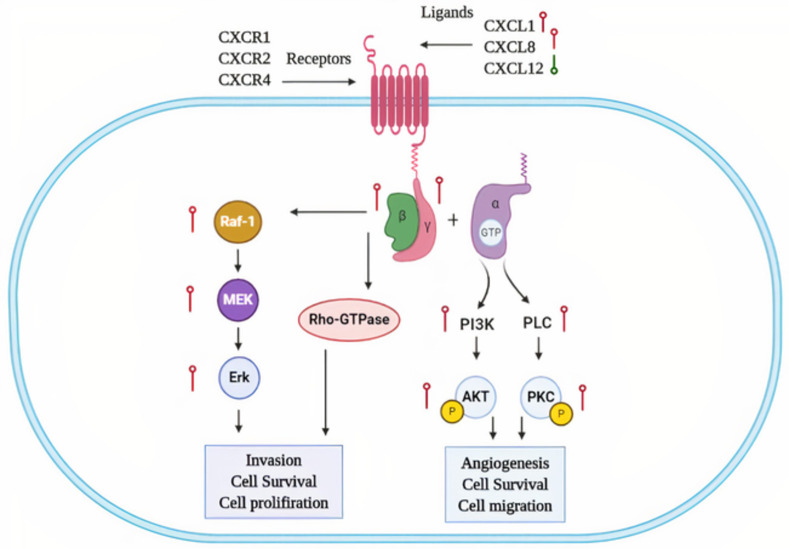
Crosstalk between the real hub genes involved pathways. At the cellular level, CXCL1, CXCL8 or CXCL12 binds to the GPCRs (CXCR1, CXCR2, or CXCR4) and activates the G protein. In this study, the up-regulation of GPCRs ligands (CXCL1 and CXCL8) and G protein subunits (β and γ) collectively are supposed to up-regulate the various downstream pathways. For example, the Heterotrimeric Gα subunit up-regulation in this study further up-regulates its main effectors PLC and PI3K to induce hyper phosphorylation of PKC and Akt, respectively. The oncogenic roles of these two signaling pathways have already been reported to activate various respective transcription factors associated with angiogenesis, cell survival, and migration of tumor cells. On the other hand, β and γ G protein subunits up-regulation in this study further up-regulates the Rho-GTPase family and Raf-1/MAP/Erk signaling cascade which has been earlier reported to contribute in the cell invasion, cell survival, and cell proliferation [78].

The real hub gene Chemokine (C-X-C motif) ligand 1 (CXCL1), also known as GRO-α, belongs to the G protein-coupled receptor family that specifically binds to the CXC chemokine receptor 2 which activate the RAS (Rat sarcoma) pathway in cell proliferation [74]. Different previous studies have demonstrated the role of CXCL1’s up-regulation in CRC metastasis and progression [75–77], however, none of these studies generalized CXCL1 in CRC patients of different clinicopathological features (different races, cancer stages, genders, age groups, and body weights). But this study identified the significant (p<0.05) up-regulation of CXCL1 mRNA in COAD patients of different races, cancer stages, genders, age groups, and body weights. Moreover, results of this study also showed the up-regulation of CXCL1 at translation level in COAD patients relative to controls. The correlation analysis between the CXCL1 expression and methylation status revealed the expected significant (p<0.05) negative correlation which strengthened the role hypomethyltion in the up-regulation of CXCL1. Lastly, the results of CXCL1 genetic alteration analysis revealed that CXCL1 expression is unlikely to be the effect of genetic alterations as alterations were noticed in a very small proportion (0.4%) of the COAD patients. Collectively, our data suggested CXCL1 up-regulation as a novel potential diagnostic biomarker in CRC patients of different races, cancer stages, genders, age groups, and body weights. The details of crosstalk between the real hub genes involved pathways in context to CRC is given in Fig 17.

The OS survival analysis of the real hub genes demonstrated that high expression of GNG4 was the good prognostic biomarkers while CXCL12, CXCL8, AGT, GNB1, and CXCL1 were the bad prognostic biomarkers for predicting the OS duration of COAD patients.

To further clarify the underlying mechanisms of real hub genes in CRC tumorigenesis, we performed the correlation analysis between the real hub genes expression and CD8+ T immune cells’ infiltration in COAD. The CD8+ T immune cells are known as the major drivers of the anticancer immunity [79] and earlier, CD8+ T immune cells infiltration was utilized as a diagnostic marker for the early detection of laryngeal squamous cell carcinoma abbreviated as LSCC [80]. Furthermore, *Trojan et al*. have also successfully used CD8+ T immune cells’ infiltration for the personalized immunotherapy trials in LSCC [81]. Our results revealed a significant (p>0.05) positive correlation between the mRNA expression levels of the CXCL12, GNB1 and CXCL1 and CD8+ T immune cells’ infiltration while a significant (p>0.05) negative correlation between the mRNA expression of CXCL8, AGT and GNG4 and CD8+ T immune cells’ infiltration. Taken together, these correlations have highlighted the new aspect of the real hub genes in CRC tumorigenesis via regulating CD8+ T immune cells’ infiltration. Best to our knowledge, this study is the first study to investigate the spearman correlation coefficient between the expression of real hub genes (CXCL12, CXCL8, AGT, GNB1, GNG4, and CXCL1) and CD8+ T immune cells’ infiltration in CRC. These correlations may bring new ideas for the treatment of CRC patients who do not benefit from the existing immune checkpoint inhibitors/regulators.

The miRNAs are the small non-coding RNA molecules (~22 nucleotides) responsible for the degradation and translation of the mRNAs in plants and animals [82]. In CRC, conflicting reports were found reporting expression variations in miR-1-3p. Mainly, miR-1-3p was found down-regulated in different studies investigating association of miR-1-3p with CRC [47–49]. However, one exceptional study has also reported its up-regulation [50]. In view to the results of present study, we speculate that four real hub genes (CXCL8, CXCL12, CXCL1, and GNB1) function as an oncogenes and down-regulation of mir-1-3p may lead to the up-regulation of these genes as a different axis (mir-1-3p/CXCL8 or CXCL12, CXCL1, and GNB1) in the pathogenesis of CRC. Best to our knowledge, this study is the first study to report the tumorigenic role of mir-1-3p together with CXCL8, CXCL12, CXCL1, and GNB1 in CRC. In addition, we have also identified various drugs (Fig 16) that could be used in the treatment of CRC by regulating the expression of real hub genes.

Some worth monitoring limitations of the present study are inevitable. Firstly, the lack of ample data regarding expression status of real hub genes in CRC patients of Pakistani population might be one limitation. Secondly, we only considered the top six real hub genes, however, the remaining genes needed to be considered in the future experiments. Finally, the information related to various clinical parameters of COAD patients like family history of cancer, tumor location, smoking, and alcohol drinking history is not available on the utilized databases and thus has not been analyzed in detail and discussed in the manuscript.

## 5. Conclusion

In summary, we have identified the a panel of six differentially expressed real hub genes (hub genes of the hub genes) including CXCL12, CXCL8, AGT, GNB1, GNG4, and CXCL1 and their underlying molecular pathways that could be employed as a possible diagnostic and prognostic biomarkers in CRC patients of different races, cancer stages, genders, age groups, and body weight and thus may help to overcome the heterogeneity-specific barrier. However, prior to clinical implementation there is need to launch extensive biological investigations especially for the under-represented populations in the expression datasets used during present study.

## Supporting information

S1 Table Row list of the CRC-associated hub genes extracted from the previous studies. (DOCX)
