# Supplementary material for: Novel diagnostic and prognostic biomarkers of colorectal cancer: Capable to overcome the heterogeneity-specific barrier and valid for global applications
Source: PLoS One. 2021 Sep 2;16(9):e0256020. doi: 10.1371/journal.pone.0256020 (PMC8412268; doi:10.1371/journal.pone.0256020)
Supplement: S1 Table — (DOCX) [file pone.0256020.s001.docx]

| **Datasets** | **Name of hub genes** | **No. hub genes** | **Reference** |
| --- | --- | --- | --- |
| GSE17538 GSE29623 | HCLS1, EVI2B, CD48 | 03 | [1-21] |
| GSE81558 GSE21510 | DSN1, ALDH1A1, TUBAL3, RRM2, SHMT2, PGM1, CCT6A, IDH3A, HSPA2, UGP2, RUVBL1, CDK1, CCNB1, MAD2L1, AHCY, HSPH1, BUB1B, DARS, KIF18A, NUF2, CENPF, ERCC6L, SPC25 | 23 |  |
| GSE44076 | CXCL1, CXCL12, SST, NPY, PPY, LPAR1, CCL19, GNG4, CXCL5, CXCL2, CXCL3, NPY1R, CCL28, TIMP1, SPP1, MMP3, GCG, KIT, BMP2, COL1A1 | 20 |  |
| GSE24514 GSE32323 GSE110225 GSE113513 | CXCL1, CXCL3, CXCL8, CXCL11, NMU, PPBP | 06 |  |
| GSE20916 GSE73360 GSE44861 | COL3A1, TGFR1, COL1A1, CD44, SPP1, MMP1. SPARC, MYC, COL1A2, TIMP1, MMP3, CA2, CXCL1, SERPINB5, CCND1, PTGS2, MET, GDF15, ECT2, CKAP2, CSE1L, ABCE1, RFC3, TPx2, SLC4A4, SLC26A3, CLCA4, SLC25A2, CEACAM7. | 29 |  |
| GSE23878 | PDGFRB, MET, FZD2, CCND1, PRKCB, ARHGEF6, JUP, WNT2, WNT5A, WNT11 | 10 |  |
| GSE41258 | PYY, CXCL3, CXCL11, CXCL8, CXCL12, CCL20, MMP3, P2RY14, NPY1R, CXCL1 | 10 |  |
| GSE110224 | CXCL8, FPR2, CCNB1, KIF2C, TPX2, CXCL1, KIF20A, DLGAP5, NCAPG, RRM2, UBE2C, EXO1, CDC45, CXCL10, DTL, PF4, SST,CEP55 | 18 |  |
| GSE35279 GSE21815 | ADNP, CCND1, CD44, CDK4, CEBPB, CENPA, CENPH, CENPN, MYC, RFC2 | 10 |  |
| GSE87211 | CXCL11, SSTR1, SSTR2, CXCL12, HCAR3, SST, APLN, CXCL8, CXCR2, PPY, NPY, PPBP, SAA1, PMCH, GAL, CXCR1, NPY1R, CCL23, CXCL6, CCL28, FPR2, CXCL1, CXCL2, CXCL3, HTR1D, GALR1, CNR1, AGT, FPR1, PTGDR2, CCR8, INSL5, F2RL2, GCC, GRP, OXTR, GPR4, NPSR1, UTS2B, PROK2, AGTR1, EDN3, CHRM1 | 43 |  |
| GSE117606 | DK1, CCNA2, TOP2A, PLK1, MAD2L1, AURKA, BUB1B, UBE2C, TPX2, RRM2, KIF11, NCAPG, MELK, NUSAP1, MCM4, RFC4, PTTG1, CHEK1, CEP55, DTL | 20 |  |
| GSE28000 GSE21815 GSE75970 | CDK1, CCNB1, CENPE, KIF20A, CXCL12, DLGAP5, CCNA2, ITGA2, MAD2L1, NMU | 10 |  |
| GSE20916 GSE39582 | BGN, SULF1, COL1A1, FA , THBS2 , CTHRC1, COL5A2, COL1A2 | 08 |  |
| GSE9348 GSE22598 GSE113513 | AQP8, CLCA4, GUCA2B, MS4A12, GUCA2A, CA2, ABCG2, CLDN8, GCG, ZG16, PKIB, CA4, BEST4, CA1, MT1M, CD177, HSD17B2, INSL5, ADH1C, CLCA1, FOXQ, KRT23, LY6G6D, MMP7, CDH3, MMP3, CST1, CRNDE, DPEP1, MMP1, EPHX4, CTHRC1, CLDN1, CEL, CLDN2, SLC35D3, COL11A1, CXCL3, SLCO1B3, CKMT2 | 40 |  |
| GSE32323 GSE74602  GSE113513 | CCL19, CXCL1, CXCL5, CXCL11, CXCL12, GNG4, INSL5, NMU, PYY, SST | 10 |  |
| GSE101502 | NUDT21, GNB1, CLINT1, COL1A2 | 4 |  |
| GSE2509 | EGFR, HRas, Wnt5a, Akt1, CDKN1a, | 05 |  |
| GSE7621 | PCNA, CCND1, NAT1, NAT2 | 04 |  |
| GSE14333 | PLAGL2, POFUT1 | 02 |  |
| GSE4183 | TOP2A, MYC, CCND1, CDK1, ACLY, VEDFA, GMPS, ENO1, CCNB1,ACTA2, AURKA | 11 |  |
| GSE4107 | CDC42, TEX11, QKI, CAV, FN1 | 05 |  |

**References**

1. Yuan Y, Chen J, Wang J, Xu M, Zhang Y, Sun P, et al. Identification Hub Genes in Colorectal Cancer by Integrating Weighted Gene Co-Expression Network Analysis and Clinical Validation in vivo and vitro. Front Oncol. 2020;10: 638-.

2. Zhou H, Yang Z, Yue J, Chen Y, Chen T, Mu T, et al. Identification of potential hub genes via bioinformatics analysis combined with experimental verification in colorectal cancer. Mol Carcinog. 2020;59.

3. Yang W, Ma J, Zhou W, Li Z, Zhou X, Cao B, et al. Identification of hub genes and outcome in colon cancer based on bioinformatics analysis. Cancer Manag Res. 2018;11: 323-38.

4. Gong B, Kao Y, Zhang C, Sun F, Gong Z, Chen J. Identification of Hub Genes Related to Carcinogenesis and Prognosis in Colorectal Cancer Based on Integrated Bioinformatics. Mediators Inflamm. 2020;2020: 5934821-.

5. Dai GP, Wang LP, Wen YQ, Ren XQ, Zuo SG. Identification of key genes for predicting colorectal cancer prognosis by integrated bioinformatics analysis. Oncol Lett. 2020;19: 388-98.

6. Wang Y, Zheng T. Screening of hub genes and pathways in colorectal cancer with microarray technology. Pathol Oncol Res. 2014;20: 611-8.

7. Wang YR, Meng LB, Su F, Qiu Y, Shi JH, Xu X, et al. Insights regarding novel biomarkers and the pathogenesis of primary colorectal carcinoma based on bioinformatic analysis. Comput Biol Chem. 2020;85: 4.

8. Wang X, Hu S, Ji W, Tang Y, Zhang S. Identification of genes associated with clinicopathological features of colorectal cancer. J Int Med Res. 2020;48: 300060520912139-.

9. Rahman MR, Islam T, Gov E, Turanli B, Gulfidan G, Shahjaman M, et al. Identification of Prognostic Biomarker Signatures and Candidate Drugs in Colorectal Cancer: Insights from Systems Biology Analysis. Medicina. 2019;55.

10. Lv J, Li L. Hub Genes and Key Pathway Identification in Colorectal Cancer Based on Bioinformatic Analysis. BioMed research international. 2019;2019: 1545680-.

11. Ding X, Duan H, Luo H. Identification of Core Gene Expression Signature and Key Pathways in Colorectal Cancer. Frontiers in Genetics. 2020;11.

12. Guo Y, Bao Y, Ma M, Yang W. Identification of Key Candidate Genes and Pathways in Colorectal Cancer by Integrated Bioinformatical Analysis. Int J Mol Sci. 2017;18: 722.

13. Wei S, Chen J, Huang Y, Sun Q, Wang H, Liang X, et al. Identification of hub genes and construction of transcriptional regulatory network for the progression of colon adenocarcinoma hub genes and TF regulatory network of colon adenocarcinoma. J Cell Physio. 2020;235: 2037-48.

14. Yu C, Chen F, Jiang J, Zhang H, Zhou M. Screening key genes and signaling pathways in colorectal cancer by integrated bioinformatics analysis. Mol Med Rep. 2019;20: 1259-69.

15. Chen L, Lu D, Sun K, Xu Y, Hu P, Li X, et al. Identification of biomarkers associated with diagnosis and prognosis of colorectal cancer patients based on integrated bioinformatics analysis. Gene. 2019;692: 119-25.

16. Dong Z, Lin W, Kujawa SA, Wu S, Wang C. Predicting MicroRNA Target Genes and Identifying Hub Genes in IIA Stage Colon Cancer Patients Using Bioinformatics Analysis. Biomed Res Int. 2019;7.

17. Qi C, Chen Y, Zhou Y, Huang X, Li G, Zeng J, et al. Delineating the underlying molecular mechanisms and key genes involved in metastasis of colorectal cancer via bioinformatics analysis. Oncol Rep. 2018;39: 2297-305.

18. Liu F, Ji F, Ji Y, Jiang Y, Sun X, Lu Y, et al. In-depth analysis of the critical genes and pathways in colorectal cancer. Int J Mol Med. 2015;36: 923-30.

19. Lv Y, Xie B, Bai B, Shan L, Zheng W, Huang X, et al. Weighted gene coexpression analysis indicates that PLAGL2 and POFUT1 are related to the differential features of proximal and distal colorectal cancer. Oncol Rep. 2019;42: 2473-85.

20. He J, Liu W. Identification of disrupted pathways associated with colon cancer based on combining protein-protein interactions and pathway data. J Cancer Res Ther. 2018;14: S998-S1003.

21. Luo T, Wu S, Shen X, Li L. Network cluster analysis of protein-protein interaction network identified biomarker for early onset colorectal cancer. Mol Biol Rep. 2013;40: 6561-8.
